# Differential regulation of two types of monogalactosyldiacylglycerol synthase in membrane lipid remodeling under phosphate-limited conditions in sesame plants

**DOI:** 10.3389/fpls.2013.00469

**Published:** 2013-11-19

**Authors:** Mie Shimojima, Takahide Watanabe, Yuka Madoka, Ryota Koizumi, Masayuki P. Yamamoto, Kyojiro Masuda, Kyoji Yamada, Shinji Masuda, Hiroyuki Ohta

**Affiliations:** ^1^Center for Biological Resources and Informatics, Tokyo Institute of TechnologyYokohama, Japan; ^2^Graduate School of Biological Sciences, Tokyo Institute of TechnologyYokohama, Japan; ^3^Graduate School of Science and Engineering, University of ToyamaToyama, Japan; ^4^Department of Biology, Faculty of Science, University of ToyamaToyama, Japan; ^5^Earth-Life Science Institute, Tokyo Institute of TechnologyTokyo, Japan; ^6^Core Research for Evolutional Science and Technology, Japan Science and Technology AgencyTokyo, Japan

**Keywords:** galactolipid, monogalactosyldiacylglycerol, phosphate deficiency, sesame

## Abstract

Phosphate (Pi) limitation causes drastic lipid remodeling in plant membranes. Glycolipids substitute for the phospholipids that are degraded, thereby supplying Pi needed for essential biological processes. Two major types of remodeling of membrane lipids occur in higher plants: whereas one involves an increase in the concentration of sulfoquinovosyldiacylglycerol in plastids to compensate for a decreased concentration of phosphatidylglycerol, the other involves digalactosyldiacylglycerol (DGDG) synthesis in plastids and the export of DGDG to extraplastidial membranes to compensate for reduced abundances of phospholipids. Lipid remodeling depends on an adequate supply of monogalactosyldiacylglycerol (MGDG), which is a substrate that supports the elevated rate of DGDG synthesis that is induced by low Pi availability. Regulation of MGDG synthesis has been analyzed most extensively using the model plant *Arabidopsis thaliana*, although orthologous genes that encode putative MGDG synthases exist in photosynthetic organisms from bacteria to higher plants. We recently hypothesized that two types of MGDG synthase diverged after the appearance of seed plants. This divergence might have both enabled plants to adapt to a wide range of Pi availability in soils and contributed to the diversity of seed plants. In the work presented here, we found that membrane lipid remodeling also takes place in sesame, which is one of the most common traditional crops grown in Asia. We identified two types of MGDG synthase from sesame (encoded by *SeMGD1* and *SeMGD2*) and analyzed their enzymatic properties. Our results show that both genes correspond to the *Arabidopsis* type-A and -B isoforms of MGDG synthase. Notably, whereas Pi limitation up-regulates only the gene encoding the type-B isoform of *Arabidopsis*, low Pi availability up-regulates the expression of both *SeMGD1* and *SeMGD2*. We discuss the significance of the different responses to low Pi availability in sesame and *Arabidopsis*.

## INTRODUCTION

The regulation of galactolipid synthesis in *Arabidopsis thaliana* under phosphate (Pi)-limited conditions has been studied extensively. Under Pi-sufficient conditions, the galactolipids monogalactosyldiacylglycerol (MGDG) and digalactosyldiacylglycerol (DGDG) are found exclusively and abundantly in plastids, especially in thylakoid membranes. They are not observed in other extraplastidial membranes (Joyard et al., 1998). Under Pi-depleted conditions, synthesis of MGDG and DGDG is up-regulated, and DGDG is exported from plastids to extraplastidial membranes to help maintain membrane structure after phospholipid degradation (Essigmann et al., 1998; Härtel et al., 2000; Andersson et al., 2003, 2005; Jouhet et al., 2004). DGDG can substitute for phosphatidylcholine (PC) in the membrane because DGDG and PC are both bilayer-forming lipids, whereas MGDG is not (Murphy, 1986). Regarding the substitution for non-bilayer or anionic phospholipids such as phosphatidylethanolamine and phosphatidylinositol under Pi-depleted conditions, glucuronosyldiacylglycerol was suggested to play an essential role (Okazaki et al., 2013).

Although MGDG moiety is unlikely to have a role in PC substitution, MGDG plays an important role as a precursor for DGDG synthesis under Pi-depleted conditions. DGDG is produced by galactosylation of MGDG by the DGDG synthases DGD1 and DGD2 in *Arabidopsis* (Härtel et al., 2000; Kelly and Dörmann, 2002; Kelly et al., 2003). Both DGD1 and DGD2 contribute to DGDG production under Pi-sufficient and Pi-limiting conditions, but the contribution of DGD2 to the increase in extraplastidial DGDG concentrations under Pi-depleted conditions is larger than that of DGD1 (Härtel et al., 2000; Kelly and Dörmann, 2002; Kelly et al., 2003).

There are three MGDG synthases in *Arabidopsis* that are classified into two types: type A (MGD1) and type B (MGD2 and MGD3; Awai et al., 2001). Type-A MGD1 is mainly involved in the bulk of MGDG synthesis and plays a critical role during the development of chloroplasts and the formation of the photosynthetic apparatus. Analysis of *mgd1-1* knockdown and *mgd1-2* knockout mutants indicated that the role of MGD1 cannot be fully complemented by MGD2 or MGD3 (Jarvis et al., 2000; Kobayashi et al., 2007, 2013). Under nutrient-sufficient conditions, the expression levels of *MGD2* and *MGD3* are very low in vegetative tissues (Awai et al., 2001; Kobayashi et al., 2004). The relatively high levels of *MGD2* transcripts in non-green tissues such as flowers suggest that galactolipids are likely to have important roles in these organs (Awai et al., 2001; Kobayashi et al., 2004). Indeed, DGDG is a major glycolipid in floral organs, and galactolipid-producing activity is highly up-regulated in the pistils, petals, and elongated pollen tubes of *Petunia* *hybrida* and *Lilium* *longiflorum* flowers during their development (Nakamura et al., 2003, 2009a). However, the role of type-B MGDG synthase during flower development has not been defined given that neither a *mgd2* single nor a *mgd2mgd3* double knockout mutant displayed any distinctive flower phenotype compared with that of wild-type (WT) plants (Kobayashi et al., 2009a). This suggests that type-A MGDG synthase may complement the function of type-B MGDG synthase in the flower (Kobayashi et al., 2009a). Under nutrient-sufficient conditions, *AtMGD3* is relatively highly expressed in roots compared with other organs, but roots of *mgd3* plants showed no obvious phenotype compared with WT. These results suggest that type-B MGDG synthases have no crucial role in seedling development under nutrient-sufficient conditions, at least in *Arabidopsis*.

In contrast, under Pi-depleted conditions, type-B MGDG synthases have an essential role in membrane lipid remodeling (Kobayashi et al., 2009a). MGD2 and MGD3 are main contributors to the increase in DGDG concentrations during Pi starvation by supplying a substrate for DGDG synthesis. Although levels of *MGD1* transcripts remain unchanged under Pi-depleted conditions, MGD1 also partially contributes to the increase in DGDG concentrations under Pi-depleted conditions; concentrations of DGDG in leaves are increased in *mgd2mgd3* under Pi-depleted conditions (Kobayashi et al., 2009a). Moreover, among the two type-B MGDG synthases of *Arabidopsis*, MGD3 seems to be a main contributor of MGDG synthesis induced by low Pi availability; although developmental defects are observed in *mgd3* and *mgd2mgd3* mutants, no significant defects are observed in the *mgd2* mutant (Kobayashi et al., 2009a).

More than a decade has passed since the first isolation of genes encoding type-B MGDG synthases from *Arabidopsis*, and molecular and biochemical analyses of *Arabidopsis* mutant plants or WT plants treated with chemical inhibitors of MGDG synthesis have revealed many details of the regulation of MGDG synthesis under Pi-depleted conditions (Botté et al., 2011; Boudière et al., 2012). Besides *Arabidopsis*, membrane lipid remodeling under Pi-depleted conditions has also been observed in *Avena sativa, Glycine max, Phaseolus vulgaris, Acer pseudoplatanus* (suspension-cell cultures), *Helianthus annuus, Oryza sativa, Raphanus sativus, Tropaeolum majus, Zea mays*, and six Proteaceae species (Andersson et al., 2003; Gaude et al., 2004; Jouhet et al., 2004; Russo et al., 2007; Tjellström et al., 2008; Lambers et al., 2012). These data suggest that the up-regulation of galactolipid synthesis following Pi depletion might be conserved in several higher plants and that it might offer a competitive advantage that allows certain plant species to survive in Pi-limited environments (Kobayashi et al., 2009b; Yuzawa et al., 2012). Indeed, recent comprehensive phylogenetic analyses of genes that encode MGDG synthases in bacteria and plants conducted by our group suggested that a gene encoding a type-B MGDG synthase might have been acquired around the time of the emergence of seed plants; this gene might have been critical to the adaptation of plant species to Pi-limited conditions, which may often have occurred on land during an early phase of the evolution of life on earth (Ohta et al., 2012).

Here we report our findings following analysis of the regulation of MGDG synthesis in sesame, *Sesamum indicum* L. Sesame is one of the most popular traditional crops in Asia. Sesame plants can grow in soils such as acidic volcanic ash, which suggests their potential suitability as model plants that are relatively tolerant to low available Pi. In this paper, we mainly compared the regulation of MGDG synthesis in sesame with that in *Arabidopsis* because the regulation of MGDG synthesis at a molecular level is better understood in *Arabidopsis* than in any of the other higher plants in which this has been analyzed.

## RESULTS

### MORPHOLOGICAL DIFFERENCES BETWEEN SESAME AND *ARABIDOPSIS* UNDER Pi-DEPLETED CONDITIONS

**FIGURE 1:**
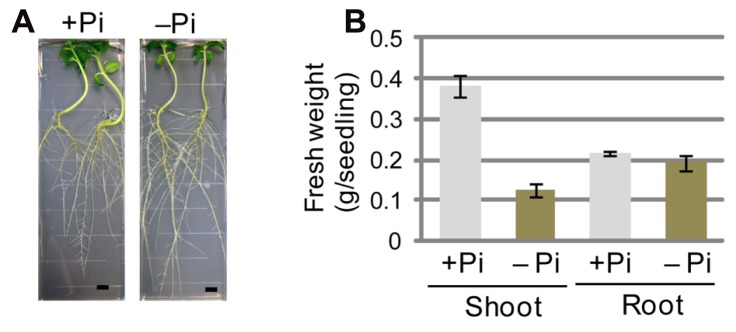
**Sesame plants grown under phosphate (Pi)-sufficient and Pi-depleted conditions.** **(A)** Growth phenotypes of sesame seedlings grown on Pi-sufficient medium for 5 days and then on Pi-sufficient (+Pi) or Pi-depleted (-Pi) medium for 7 days. Bars represent 1 cm. **(B)** Fresh weights of shoots and roots of sesame plants grown on Pi-sufficient medium for 5 days and then on +Pi or -Pi medium for 14 days. Values shown for each of shoots (*n* = 3) and roots (*n* = 3) represent the mean ± SE for three independent measurements.

Morphology and biochemical processes can be markedly affected when plants are exposed to a Pi-depleted growth environment. Morphological changes, such as growth retardation of shoots and primary roots and elongation of lateral roots and root hairs, have been documented extensively in *Arabidopsis* plants transferred to Pi-depleted conditions (Bates and Lynch, 1996; Raghothama, 1999; Ticconi and Abel, 2004; Desnos, 2008; Péret et al., 2011; Sato and Miura, 2011). When sesame seedlings were grown under Pi-depleted conditions, morphological changes in the lateral roots seemed to be more obvious than those observed in *Arabidopsis* (**Figure 1A**). We measured fresh weight per seedling under Pi-sufficient or -depleted growth conditions for shoots and roots, respectively (**Figure 1B**). It should be noted that conflicting effects of Pi depletion have been reported for *Arabidopsis*. Whereas Kobayashi et al. (2009a) reported that the fresh weight of shoots and that of roots both decreased when plants were grown under Pi-depleted conditions (0 mM Pi in agar medium containing 1% sucrose), Versaw and Harrison (2002) observed that the fresh weight of shoots decreased but that of roots was unchanged under other Pi-depleted conditions (0.2 mM Pi in agar medium without sucrose). Sucrose in the growth medium enhances the sensitivity of plants to Pi depletion (Lei and Liu, 2011). Given that we used the same growth medium as Kobayashi et al. (2009a), we compared the sesame phenotype with their *Arabidopsis* data.

In sesame, fresh weight of shoots was drastically decreased upon transfer to Pi-depleted medium, similar to the effects shown by Kobayashi et al. (2009a) for *Arabidopsis*. In contrast, the fresh weight of roots remained unchanged upon transfer to Pi-depleted medium (**Figure 1B**). These data showed that, when sesame and *Arabidopsis* are compared, the morphological changes caused by low-Pi stress are similar in shoots but different in roots. The data further suggested that the significant elongation of lateral roots in sesame plants grown under Pi-depleted conditions might offer an advantage for survival under conditions of low Pi availability.

### COMPARISON OF THE MEMBRANE LIPID COMPOSITIONS OF SESAME AND *ARABIDOPSIS* DURING Pi STARVATION

**FIGURE 2:**
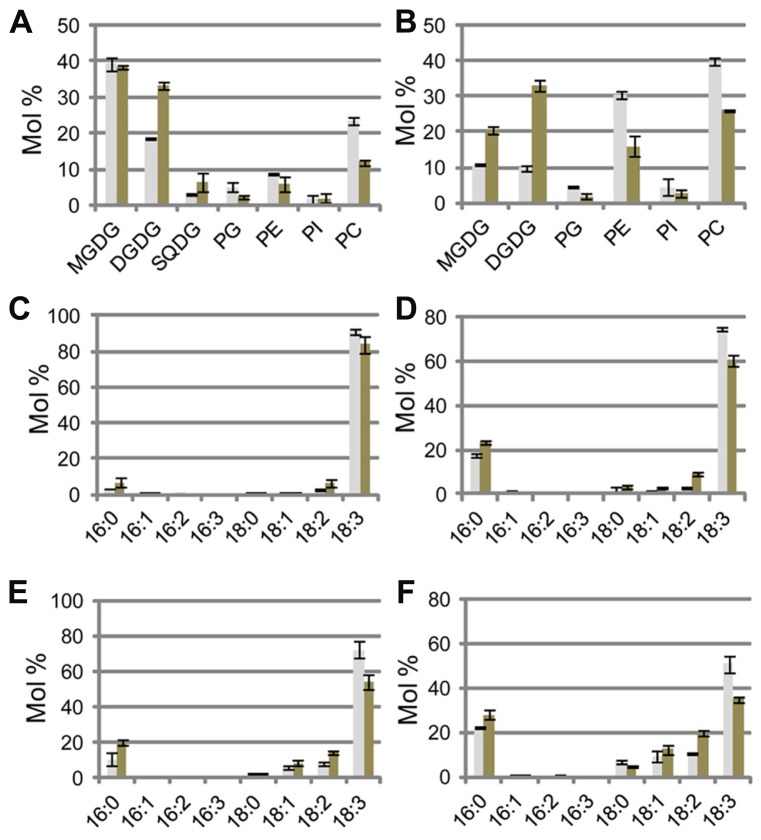
**Lipid analysis of sesame seedlings under phosphate (Pi)-sufficient and Pi-depleted conditions.** **(A,B)** Molar ratio of membrane lipids of shoots **(A)** and roots **(B)**. Fatty acid compositions of MGDG **(C,E)** and DGDG **(D,F)** in shoots **(C,D)** and roots **(E,F)**. Gray bars and black bars indicate Pi-sufficient and Pi-depleted conditions, respectively. MGDG, monogalactosyldiacylglycerol; DGDG, digalactosyldiacylglycerol; PC, phosphatidylcholine; PE, phosphatidylethanolamine; PG, phosphatidylglycerol; PI, phosphatidylinositol; SQDG, sulfoquinovosyldiacylglycerol. Values represent the mean ± SD from three independent measurements.

Regarding the lipid compositions of cellular membranes under Pi-depleted conditions, *Arabidopsis* is known to degrade phospholipids and supply Pi needed for essential biological processes. A simultaneous increase in the concentration of the galactolipid DGDG compensates for the loss of phospholipids from membranes. In both shoots and roots of sesame plants grown under Pi-depleted conditions, the mol% (relative to total membrane lipids) of DGDG and sulfoquinovosyldiacylglycerol (SQDG) increased and that of phospholipids (phosphatidylglycerol, phosphatidylethanolamine, phosphatidylinositol, and PC) decreased (**Figures 2A, B**). In shoots, most of the changes were comparable between *Arabidopsis* and sesame (**Figure 2A**). However, Pi depletion decreased the phospholipid mol% (relative to total membrane lipids) by 32% (from 79 to 47%) in sesame roots (**Figure 2B**). This reduction is slightly larger than the 24% reduction (from 88 to 64%) observed in *Arabidopsis* roots (Kobayashi et al., 2009a). In sesame roots grown under Pi-sufficient conditions, the molar ratio of galactolipids MGDG and DGDG in the total membrane lipids (20%) was much higher than that in *Arabidopsis* roots (9%; Kobayashi et al., 2009a). This difference might result from differences in the abundance of plastids in sesame roots under both of the Pi conditions tested. Exposure of *Arabidopsis* to Pi-depleted conditions increases the ratio of DGDG to total acyl lipids in roots but not that of MGDG to total acyl lipids (Kobayashi et al., 2009a). Under Pi-depleted conditions, however, not only were mol% of DGDG and MGDG in sesame roots increased relative to the respective mol% under Pi-sufficient conditions, but also the total molar ratio of MGDG and DGDG (combined) reached 53% of the total membrane lipids, which is 20% higher than that measured in *Arabidopsis* roots (Kobayashi et al., 2009a).

Phosphate depletion caused a marked decrease in the abundances of phospholipids in sesame roots. These data suggested that, under Pi-depleted conditions, MGDG synthesis in sesame roots was more strongly up-regulated than in *Arabidopsis* roots and that the increased supply of Pi from the accelerated degradation of phospholipids might render sesame plants tolerant to low Pi availability by enabling them to maintain root growth under Pi-depleted conditions.

The effects of Pi depletion on the fatty acid composition of MGDG and DGDG in sesame are shown in **Figures 2C–F**. Given that MGDG contains no 16:3 fatty acid, which is a signature of “16:3 plants,” sesame should clearly be categorized as one of the “18:3 plants,” which only possess eukaryotic pathways for lipid synthesis (**Figures 2C, D**; Heinz and Roughan, 1983; Li-Beisson et al., 2013). Fatty acid compositions of DGDG under both of the Pi conditions tested were similar in sesame (**Figures 2E, F**). Given that DGDG is mainly synthesized from diacylglycerol derived from eukaryotic pathways under both Pi-sufficient and -depleted conditions, the fatty acid composition of DGDG does not differ substantially between *Arabidopsis* and sesame, although they are distinctly categorized as “16:3 plants” and “18:3 plants,” respectively (**Figures 2E, F**; Kobayashi et al., 2009a).

### ISOLATION OF THE TWO GENES THAT ENCODE SESAME MGDG SYNTHASES

**FIGURE 3:**
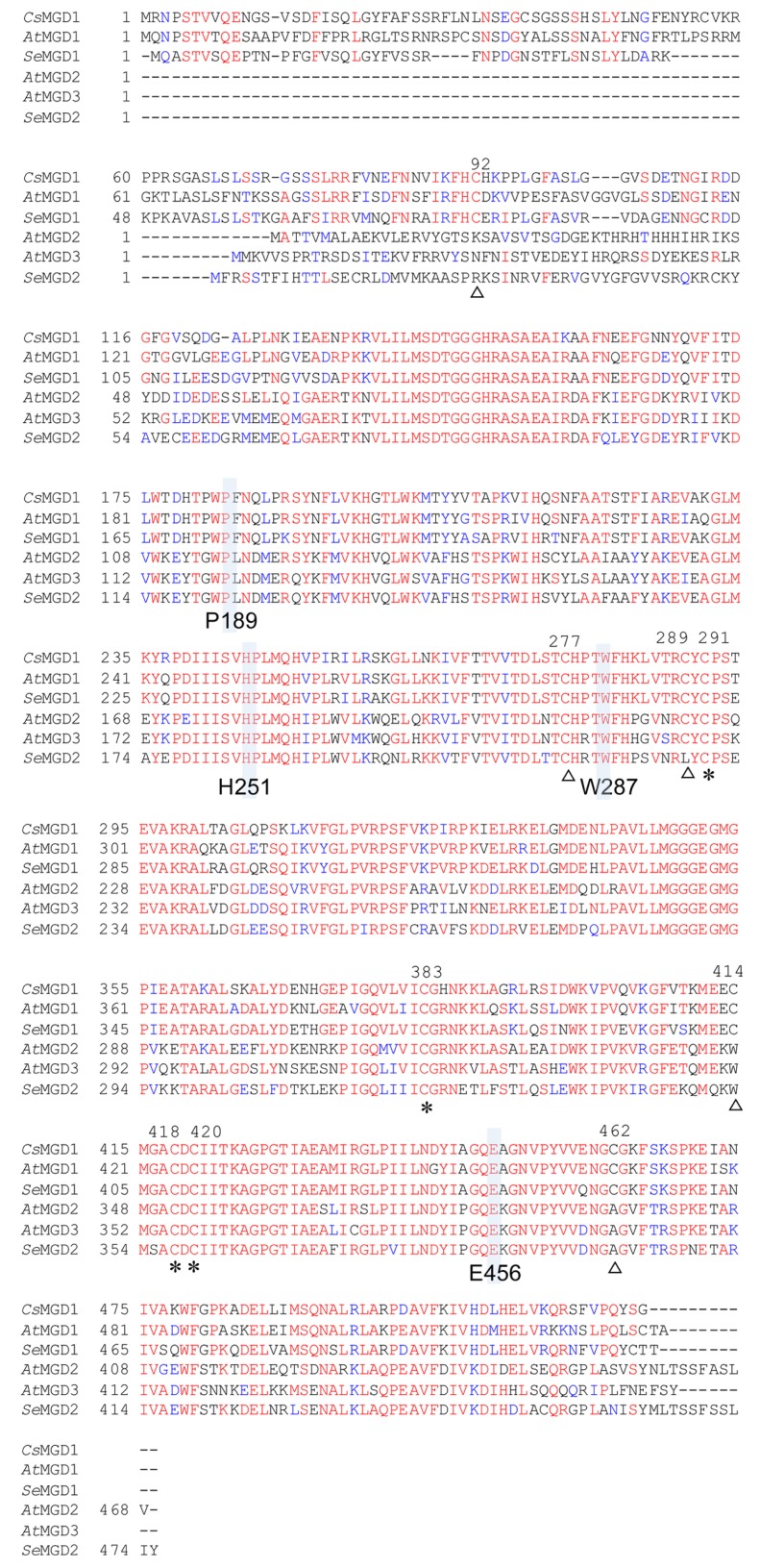
**Amino acid sequence alignment for MGDG synthases.** Red and blue indicate conserved and similar residues, respectively. Identical cysteines are indicated by asterisks; partially conserved cysteines are indicated by open triangles. Four residues in *At*MGD1 (P189, H251, W287, and E456) are shown in gray. *Cs, Cucumis sativus*; *At, Arabidopsis thaliana*; *Se, Sesamum indicum* L. Accession numbers are *Cs*MGD (P93115), *At*MGD1 (NP_849482), *Se*MGD1 (AB841066), *At*MGD2 (NP_568394), *At*MGD3 (NP_565352), and *Se*MGD2 (AB841067).

We isolated two genes for sesame MGDG synthase, namely *SeMGD1* and *SeMGD2* (accession numbers AB841066 and AB841067, respectively). *Se*MGD1 is a 516-amino acid residue protein that is predicted by ChloroP to contain a chloroplast transit peptide (Emanuelsson et al., 1999) and is categorized as a type-A MGDG synthase on the basis of its similarity to other type-A MGDG synthases (**Figure 3**). *Se*MGD2 is a 475-residue protein that is categorized as a type-B MGDG synthase. The failure to identify a putative chloroplast transit peptide in the *Se*MGD2 sequence suggests that, like *At*MGD2 and *At*MGD3, *Se*MGD2 localizes to the outer envelope membrane of chloroplasts. Comparison of the amino acid sequences of *Se*MGD1 and *Se*MGD2 with other MGDG synthases (**Figure 3**) indicated that *Se*MGD1 and *Se*MGD2 are highly similar to *At*MGD1 (81.3%) and *At*MGD2 (79.6%), respectively. We could not find the third gene for MGDG synthase by screening a sesame cDNA library. In *Arabidopsis, At*MGD2 and *At*MGD3 are both involved in MGDG synthesis under Pi-depleted conditions, but *At*MGD3 is rather predominantly involved in MGDG synthesis under Pi-depleted conditions (Kobayashi et al., 2009a). Moreover, differences in expression patterns in seedlings suggested that *At*MGD2 and *At*MGD3 have distinct tissue-specific roles in development, although the *mgd2* mutant did not show an apparent phenotype compared with the WT (Awai et al., 2001; Kobayashi et al., 2004). It is possible that sesame might have another type-B MGDG synthase that has another function, given that the cDNA library used was generated from cotyledons of seedlings grown under Pi-sufficient conditions.

### REGULATION OF TRANSCRIPT LEVELS OF *SeMGDs* UNDER Pi-DEPLETED CONDITIONS

**FIGURE 4:**
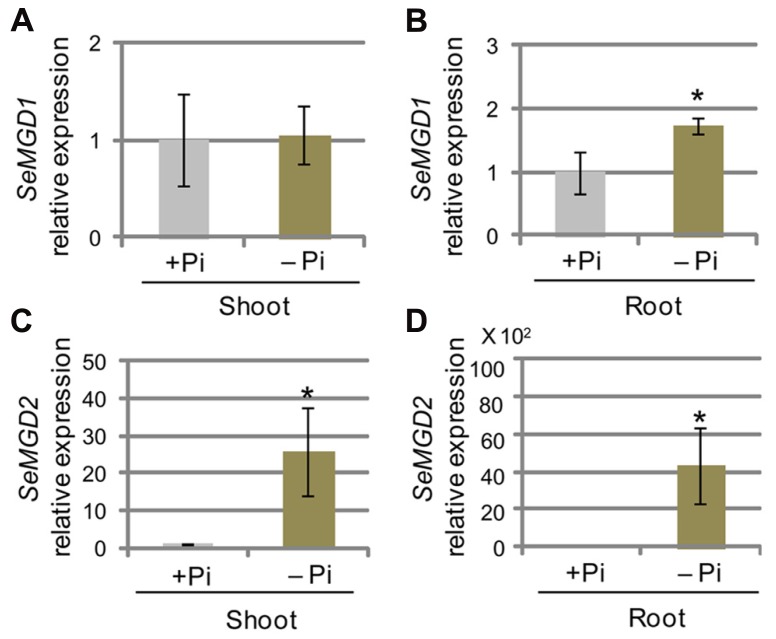
**Quantitative RT-PCR analysis of *SeMGD* expression.** **(A–D)** Relative abundances of *SeMGD1* **(A,B)** and *SeMGD2* **(C,D)** mRNAs in shoots **(A,C)** and roots **(B,D)** of plants grown under phosphate (Pi)-sufficient (+Pi) and Pi-depleted (-Pi) conditions for 14 days after 5 days of growth on Pi-sufficient medium. Values represent the mean ± SD from three independent measurements. *P < 0.05.

In *Arabidopsis, AtMGD1* is constitutively expressed, whereas the transcript abundance of *AtMGD2* and *AtMGD3* transcripts are markedly increased after transfer to Pi-depleted conditions (Awai et al., 2001; Kobayashi et al., 2004). However, it is not known whether type-B MGDG synthases from other plant species are transcriptionally up-regulated under Pi-depleted conditions. We analyzed the abundance of *SeMGD1* and *SeMGD2* mRNAs under Pi-sufficient and Pi-depleted growth conditions using quantitative RT-PCR (**Figure 4**). In sesame, the observed increases in levels of the *SeMGD2* transcript under Pi-depleted conditions both in shoots and roots (**Figures 4C, D**) showed that the transcriptional up-regulation of genes that encode type-B MGDG synthases under Pi-depleted conditions is conserved in plants other than *Arabidopsis*. Similar to what was observed for *AtMGD1, SeMGD1* expression in shoots remained unchanged under Pi-sufficient and Pi-depleted conditions (**Figure 4A**), but that in roots under Pi-depleted conditions was higher than that under Pi-sufficient conditions (**Figure 4B**). *AtMGD1* is constitutively expressed in various organs, and levels of *AtMGD1* transcript are not affected by the Pi concentration in the growth medium (Awai et al., 2001; Kobayashi et al., 2004). Thus, the regulation of MGDG synthesis under Pi-depleted conditions seems to differ between *Arabidopsis* and sesame.

### REGULATION OF *Se*MGD ACTIVITY BY REDOX STATUS

**FIGURE 5:**
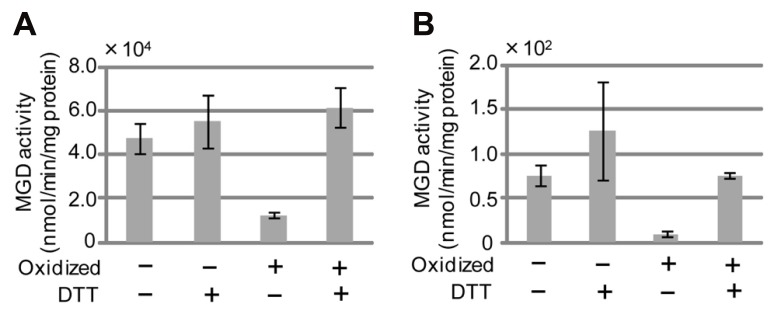
***Se*MGD activity under reducing and non-reducing conditions.** **(A)** *Se*MGD1. **(B)** *Se*MGD2. Non-oxidized, freshly purified recombinant MGDG synthases from sesame were assayed. Oxidation was performed as described in Materials and Methods. The assay was performed in the presence (+) or absence (-) of dithiothreitol (DTT). Values represent the mean ± SD from three independent measurements.

*Cucumis sativus* MGD1 (*Cs*MGD1) was the first MGDG synthase for which the gene was isolated and identified in higher plants (Shimojima et al., 1997). The enzymatic properties of *Cs*MGD1 have been well characterized (Yamaryo et al., 2003, 2006). To verify whether *Se*MGDs could be regulated by cellular redox status, we measured *Se*MGD activity under both oxidizing and reducing conditions (**Figures 5A, B**). The specific activity of *Se*MGD1 was much higher than that of *Se*MGD2, and the MGDG synthase activity of each *Se*MGD1 and *Se*MGD2 was regulated by redox status. The inactivation of both *Se*MGD1 and *Se*MGD2 by oxidization and their activation by reduction suggests that the activities of both type-A and type-B MGD might be also regulated by cellular redox status, as has been shown for *Cs*MGD1 (Yamaryo et al., 2006).

When comparing various sequences of MGDG synthases, we found five highly conserved cysteine residues at positions 277, 291, 383, 418, and 420 of *Cs*MGD1 (**Figure 3**). Given that our results showed that both types of *Se*MGDs are regulated by redox state, these five cysteines may be important for enzyme activation.

### *IN VITRO* ACTIVATION OF SESAME TYPE-B MGDG SYNTHASE BY PHOSPHATIDIC ACID

**FIGURE 6:**
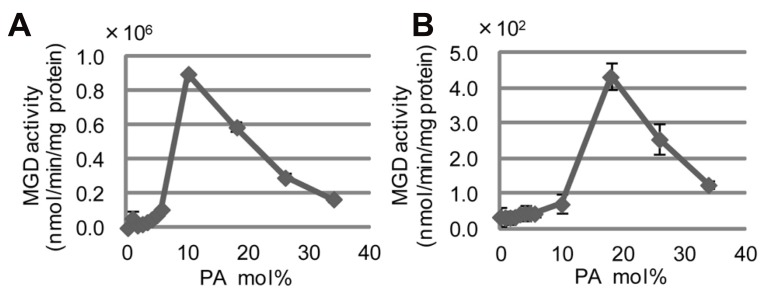
**Induction of *Se*MGD activity by phosphatidic acid (PA).** **(A)** *Se*MGD1. **(B)** *Se*MGD2. Values represent the mean ± SD from three independent measurements.

*Cs*MGD1, spinach MGD1, and *At*MGD1 are each activated *in vitro* by the inclusion of either phosphatidic acid (PA) or phosphatidylglycerol (Covès et al., 1988; Ohta et al., 1995; Dubots et al., 2010). Extensive biological analyses using *Arabidopsis* concluded that the activation of MGD1 by PA is essential and indispensable for MGDG synthesis in chloroplasts (Dubots et al., 2010). Although the PA-binding residue in the mature protein of *At*MGD1 was not determined, the important residues for full activation by PA were previously shown to be P189, H251, W287, and E456 (Dubots et al., 2010; **Figure 3**). The conservation of all four of these residues in *Se*MGD1 and *Se*MGD2, as well as the other MGDG synthases compared in **Figure 3**, suggests that both *Se*MGD1 and *Se*MGD2 might be activated by PA. All published research on the activation by lipids was performed using type-A MGDG synthases. We analyzed activation of type-A and type-B *Se*MGDs by PA using partially purified recombinant enzymes expressed in *Escherichia coli*. Intriguingly, both *Se*MGDs were activated by PA (**Figure 6**), but again the specific activity of *Se*MGD1 was much higher than that of *Se*MGD2. It should be noted that the specific activity of desalted *Se*MGD1 and *Se*MGD2 in **Figures 5A, B** (~6.0 × 10^4^ and ~0.7 × 10^2^ nmol min^-1^ mg^-1^ protein, activity of oxidized *Se*MGD1 and *Se*MGD2 + DTT, respectively) was higher than that of the non-desalted proteins in **Figures 6A, B** (~0.2 × 10^4^ and ~0.4 × 10^2^ nmol min^-1^ mg^-1^ protein, activity of *Se*MGD1 and *Se*MGD2 at PA = 0 mol%, respectively). The presence of salts could also explain the difference in PA concentrations required for the maximum activation of both types of *Se*MGD (10 mol% for *Se*MGD1, 18 mol% for *Se*MGD2) were substantially higher than those required for *At*MGD1 (0.6 mol%; Dubots et al., 2010).

## DISCUSSION

It is well known that only DGDG, but not MGDG, can substitute for membrane lipids (such as PC) outside of plastids. Given that our data indicate that MGDG accumulates in Pi-depleted roots of sesame plants, we were interested in understanding the distribution of MGDG in roots (**Figure 2B**). In *Arabidopsis*, we recently found that severe impairment of thylakoid membrane formation in *mgd1-2* is only partially complemented by the synthesis of MGDG and DGDG that is induced by low availability of Pi (Kobayashi et al., 2013). This suggests that only a small part of MGDG and DGDG produced via MGDG synthesis catalyzed by type-B MGDG synthase is able to be transported to thylakoid membranes (Kobayashi et al., 2013). We also found that up-regulation of type-B MGDG synthases under conditions of low Pi availability contributes substantially to DGDG accumulation but only slightly to MGDG accumulation (Kobayashi et al., 2013), probably because type-B MGDG synthase is located in the outer envelope membrane and the MGDG produced could be used immediately for DGDG synthesis by DGD1 and DGD2, which are also located in the outer envelope membrane of plastids. Thus, from these results, we speculate that MGDG accumulated in sesame roots might be derived from the induction of *Se*MGD1 rather than of *Se*MGD2 and that MGDG produced in Pi-deficient roots accumulates within plastids.

The level of *SeMGD1* expression was slightly increased in roots under Pi-depleted conditions although the magnitude of increase was smaller than that of *SeMGD2* in roots and shoots (**Figures 4B–D**). In *Arabidopsis, AtMGD1* mRNA abundance remains unchanged both in shoots and roots under the both Pi conditions (Awai et al., 2001; Kobayashi et al., 2004). Thus, it is possible that the increase in MGDG concentration in sesame roots was caused by a slight increase in the abundance of the transcript encoding *Se*MGD1 in roots, i.e., given that the specific activity of *Se*MGD1 was much higher than that of *Se*MGD2 (**Figures 5 and 6**). The distribution of MGDG under Pi-depleted conditions has been controversial. Härtel et al. (2000) reported that MGDG concentrations outside of plastids increase slightly under Pi-depleted conditions even in *Arabidopsis*. A slight accumulation of MGDG has been observed in the plasma membranes of oat roots under Pi-depleted conditions (Andersson et al., 2003, 2005). It will be interesting to determine where and how MGDG might accumulate in cells under Pi-depleted conditions.

Our data showed that cellular redox status can regulate the activity of both types of *Se*MGD (**Figure 5**). It has been suggested that thioredoxin is involved in the regulation of *Cs*MGD1 (Yamaryo et al., 2006). However, we have no data to show that *Se*MGD activity is regulated by thioredoxin in the same manner as *Cs*MGD1. Given that type-A MGDG synthase is localized in the inner envelope membrane of plastids, we speculate that the activation of type-A MGDG synthase by reduced thioredoxin (photosynthetic activity increases the level of reduction of the chloroplastic pool of thioredoxin) might account for the close correlation between the development of the thylakoid membrane and the maintenance of photosynthetic function. Consistent with this proposal, type-A MGDG synthase is involved in the bulk of MGDG synthesis, and the absence of the corresponding gene severely decreases photosynthetic activity (Jarvis et al., 2000; Kobayashi et al., 2007). On the other hand, the localization of type-B MGDG synthase in the outer envelope membrane of plastids might leave it less affected by the redox status of plastids (Awai et al., 2001; Kobayashi et al., 2009a). Additional research is needed to clarify how regulation of the activity of type-B MGDG synthase by cellular redox status *in vivo* might be affected by low Pi availability.

Both types of *Se*MGD were activated by PA *in vitro*, although the overall specific activity and the required concentration of PA differed markedly between *Se*MGD1 and *Se*MGD2 (**Figure 6**). *Se*MGD1 was markedly activated by 10 mol% PA, whereas *Se*MGD2 activation required 20 mol% PA. We do not know why the activation of MGDG synthases by PA displays such a narrow optimum curve, but these concentrations of PA in cell membranes (especially 20 mol% PA) are not physiologically relevant. Moreover, even after activation by PA, the activity of *Se*MGD2 was much lower than that of *Se*MGD1 (**Figure 6**). Therefore, it seems possible that each *Se*MGD is activated by PA *in vivo* through direct interaction with a PA-producing enzyme.

One unsolved problem is the pathway of PA supply for the activation of MGDG synthases. PA could be supplied either from inside or outside of plastids (Li-Beisson et al., 2013). Among the known pathways for PA supply, the only one that might be regulated by Pi depletion is the pathway that enables PA accumulation through up-regulation of phospholipase ζ2 (PLDζ2) abundance during Pi deficiency (Cruz-Ramírez et al., 2006; Acevedo-Hernández et al., 2012). PLDζ2 localizes to tonoplasts under Pi-sufficient conditions, but it has been observed that PLDζ2-enriched tonoplast domains are preferentially positioned close to mitochondria and adjacent to chloroplasts (Yamaryo et al., 2008). This suggests that the PA produced might activate MGD synthase in the envelope membrane of plastids. Localization of *Se*MGD2 in the outer envelope membrane, as is the case for *Arabidopsis* type-B MGDG synthases, might promote the interaction of *Se*MGD2 with other PA-producing proteins outside of plastids (such as PLDζ2) because the outer envelope membrane has multiple contact sites with extraplastidial membranes.

The PA pool under Pi-depleted conditions is smaller than that under Pi-sufficient conditions; this is because PA is immediately degraded to diacylglycerol by PA phosphohydrolase 1 and 2 to supply *Arabidopsis* cells with Pi (Nakamura et al., 2009b). Thus, although it remains uncertain whether *Se*MGDs are activated *in vivo* by the cellular pool of PA, it is also possible that these enzymes are activated by other lipids such as phosphatidylglycerol (Dubots et al., 2010). It remains to be resolved whether PA might be a key factor for the activation of type-B MGDG synthase. Several factors besides the availability of Pi might regulate type-B MGDG synthase at the levels of transcript abundance and protein activity *in vivo*. Kobayashi et al. (2006) evaluated the effect of phosphite on the expression of *AtMGD2* and *AtMGD3*. Phosphite, which is an inactive analog of the Pi anion, mimics Pi in signaling pathways. The results showed that increases in the abundances of *AtMGD2* and *AtMGD3* transcripts was not mediated by the damage to plants induced by a decrease in intracellular Pi. Instead, the increased abundances of *AtMGD2* and *AtMGD3* transcripts were regulated directly by a Pi-sensing system (Kobayashi et al., 2006).

All of the studies mentioned above have been performed using *Arabidopsis*. The present study did not involve a similar comparative analysis between sesame and *Arabidopsis*, but it will be the subject of future work to investigate the key regulator(s) responsible for the activation of type-B MGDG synthase under Pi-depleted conditions and to compare the regulatory mechanisms used by higher plants and how they confer tolerance of conditions of limited Pi availability. Using comparative phylogenetic analysis of MGDG synthases, we recently suggested that higher plants developed the capacity for galactolipid synthesis following the acquisition of type-B MGDG synthases on the outer envelope membrane of plastids almost 320 million years ago, which is thought to be immediately after the emergence of Spermatophyta (seed plants; Ohta et al., 2012; Yuzawa et al., 2012). Type-B MGDG synthase mainly supplies MGDG as a precursor for DGDG synthesis under Pi-depleted conditions to facilitate lipid remodeling (Kobayashi et al., 2009a, 2013). This suggests that the acquisition of type-B MGDG synthase may have been one of the key factors that promoted the modern diversity of seed plants on land given that available Pi was likely to become more scarce in some soil types than it was in seawater. Thus, it might be important to use a broad range of plant species to analyze the correlation between the expression level or enzymatic properties of type-B MGDG synthases and the Pi concentration in any given plant species’ indigenous habitat. It is also possible that the efficiency of lipid remodeling might have affected species’ survival in Pi-limited environments. Thus, comparative analyses of MGDG synthesis among diverse land plants might shed light on the evolution of seed plants.

## MATERIALS AND METHODS

### PLANT MATERIAL AND GROWTH CONDITIONS

Sesame seedlings (*S. indicum* L. strain 4294) were grown on Murashige and Skoog medium solidified with 0.8% (w/v) agar containing 1% (w/v) sucrose for 5 days at 28°C under continuous white light, transferred to Pi-sufficient (1.0 mM) or Pi-depleted (0 mM) medium, and grown for another 14 days (Härtel et al., 2000).

### LIPID ANALYSIS

Total lipids were extracted and separated by two-dimensional thin layer chromatography as described by Kobayashi et al. (2007). Lipids isolated from silica gel plates were methylated, and fatty acid methyl esters were quantified by gas chromatography using pentadecanoic acid as an internal standard (Kobayashi et al., 2006).

### CONSTRUCTION OF A cDNA LIBRARY

Total RNA was isolated from sesame cotyledons using an RNA isolation kit (Stratagene). A sesame cDNA library was generated using a cDNA synthesis kit, ZAP-cDNA synthesis kit, and ZAP-cDNA Gigapack III gold cloning kit (Stratagene).

### ISOLATION OF GENES ENCODING A SESAME MGDG SYNTHASE

Consensus sequences of type-A MGDG synthase (nucleotide number 540–1378 of *AtMGD1* ORF, NM 119327) and type-B MGDG synthase (nucleotide number 527–1184 of *AtMGD2* ORF, NM 122048) were selected based on the nucleotide sequence alignment of the sequences encoding MGDG synthases from *C. sativus* (U62622), *Nicotiana tabacum* (AB047476), *O. sativa* (AK243359, AB112060, NM 001068022), *Spinacia oleracea* (AJ249607), and *G. max* (AB047475). Degenerate primers for nested RT-PCR were constructed; for type-A MGDG synthases: forward 5′-tttntggncngancanacnccntggcc-3′ and reverse 5′-cnttnccngcntcntgnccngcnatgta-3′ for the first PCR and forward 5′-ccngatatnatnatcagtgtncatcc-3′ and reverse 5′-gnccngccttngtnatnatncantcaca-3′ for the second PCR; for type-B MGDG synthases: forward 5′-gngtncanccnntnatgcaacanattcc-3′ and reverse 5′-gcnccattntnnacnacatanggnacatt-3′ for the first PCR and forward 5′-ganctnannacntgncancctacntggtt-3′ and reverse 5′-tgnnatnatgcantcacangcncccat-3′ for the second PCR. These PCR fragments were used to screen the sesame cDNA library. The full-length *SeMGD1* cDNA was obtained by 5′-RACE using the 5′-Full RACE Core Set (TaKaRa), and that of *SeMGD2* cDNA was obtained by 5′-RACE and 3′-RACE using the RNA PCR kit (AMV) Ver. 3.0 (TaKaRa).

### QUANTITATIVE RT-PCR

Total RNA was isolated from three independent sesame samples of shoot and root using the SV Total RNA isolation system (Promega). Reverse transcription was performed using the PrimeScript RT reagent kit (TaKaRa). cDNA was amplified using SYBR PreMix Ex Taq (TaKaRa). Signal detection and quantification were performed in duplicate using the Thermal Cycler Dice Real Time System (TaKaRa). Quantification of *SeMGD1* and *SeMGD2* transcripts by quantitative PCR was carried out using the *SiACT* (JP631637) and *SiUBQ6* (JP631638) transcript levels for normalization, respectively (Wei et al., 2013). Expression levels were obtained from at least three replicates. The following gene-specific primers were used:

SeMGD1_fw: 5′ GGTCTATGGCCTTCCCGTACGG 3′

SeMGD1_rv: 5′ TGACTGCAATTTGCTGGCTAGC 3′

SeMGD2_fw: 5′ CTAGATGGGCTCGAAGAATCTC 3′

SeMGD2_rv: 5′ GATAATCAGTTGTCCGATTGGC 3′

SiACT_fw: 5′ GACGAGCTCTGCTGTAGAGAAG 3′

SiACT_rv: 5′ CCACCACTGAGAACAATATTGC 3′

SiUBQ6_fw: 5′ GATAGGCACTACTGTGGTAAGTG 3′

SiUBQ6_rv: 5′ GGAACAGCAATGGTGTCAGCAAC 3′.

### PROTEIN EXPRESSION OF *Se*MGD1 AND *Se*MGD2 IN *E. coli*

*SeMGD1* and *SeMGD2* were amplified by PCR using a gene-specific primer set (forward primer 5′-gccatatggttgatgccggggagaataa-3′ and reverse primer 5′-gcgaattcgtagttgtacaatactgaggtact-3′ for *SeMGD1*; forward primer 5′-gccatatgatgttcagaagctctacatttat-3′ and reverse primer 5′-gcgaattcgtgtaaattaggctcgagaagga-3′ for *SeMGD2*) and were then each cloned separately into the pET24a vector. Vectors were transformed into competent cells of *E. coli* strain BL21(DE3). Transformed cells were grown in Luria–Bertani medium at 37°C for 16 h and diluted 10-fold using medium to grow for a further 2 h. Protein expression was induced using 1 mM isopropyl thiogalactopyranoside for 3 h at 37°C for *Se*MGD1 and at 20°C for *Se*MGD2. Cells were collected by centrifugation at 3000 × *g* for 15 min and suspended in lysis buffer (0.1 M MOPS–NaOH, pH 7.8).

### PURIFICATION OF *Se*MGD1 AND *Se*MGD2 PROTEINS

Transformed *E. coli* were resuspended in lysis buffer and disrupted by sonication (Ultrasonic disrupter UD-201, TOMY). Cell lysates were centrifuged at 5000 × *g* for 3 min at 4°C, and the resulting supernatant was centrifuged again at 125,000 × *g* for 30 min at 4°C. Each supernatant was applied to a nickel-charged resin (Ni-NTA Agarose, Qiagen GmbH), Recombinant MGDG synthase was allowed to bind to the resin at 4°C for 30 min, and the resin was washed three times with a three-bed volume of lysis buffer that contained 0.2 M NaCl and 10 mM imidazole. Each His-tagged MGDG synthase was eluted with lysis buffer containing 0.2 M NaCl, 10% (v/v) glycerol, and 200 mM imidazole to yield a pure protein. In all experiments, protein concentrations were determined using bovine serum albumin as the standard (Bensadoun and Weinstein, 1976).

### MEASUREMENT OF MGDG SYNTHASE ACTIVITY

MGDG synthase activity was measured by determining the amount of [4,5-^3^H]galactose incorporated into the lipid fraction (Yamaryo et al., 2003). Briefly, after pre-incubation of *Se*MGD in 190 μL of assay mixture (6.4 mM diacylglycerol in 0.01% (w/v) Tween 20, 10 mM dithiothreitol, 10 mM sodium acetate, and 0.1 M MOPS–NaOH pH 7.8) at 30°C, 10 μL of UDP-[4,5-^3^H]galactose (5.1 mM, 29 Bq nmol^-1^) was added to initiate the reaction, which was allowed to proceed at 30°C for 30 min. The reaction product was quantified using an image analyzer after thin layer chromatography or measured using a liquid scintillation counter.

### ACTIVATION OF MGDG SYNTHASE BY PA

Lipids were removed from the purified *Se*MGD preparation (1 mg mL^-1^) by stirring at 4°C for 30 min in lysis buffer that contained 24 mM lauryl dimethylamino oxide. MGDG synthase activity was then assessed after the addition of PA using the method described above. PA concentration was calculated as the mol% of total micelle concentration in the assay mixture (Maréchal et al., 1994).

### OXIDATION AND REDUCTION OF MGDG SYNTHASE

Oxidation and reduction of the *Se*MGD isoforms were performed according to Yamaryo et al. (2006) with slight modifications. Briefly, the purified MGD was treated with 50 μM CuCl_2_ for 1 h on ice. The oxidized enzyme was then subjected to gel filtration (ProbeQuant G-50 Micro Column, GE Healthcare) to remove Cu^2+^ ions and salts and was then used in assays to measure the activity of oxidized MGDG synthase. Reduced MGD was prepared by incubation with 1 mM dithiothreitol at 30°C for 30 min.

## Conflict of Interest Statement

The authors declare that the research was conducted in the absence of any commercial or financial relationships that could be construed as a potential conflict of interest.
